# Rivaling paradigms in psychiatric neurosurgery: adjustability versus quick fix versus minimal-invasiveness

**DOI:** 10.3389/fnint.2015.00027

**Published:** 2015-04-02

**Authors:** Sabine Müller, Rita Riedmüller, Ansel van Oosterhout

**Affiliations:** ^1^Charité-Universitätsmedizin Berlin, Department of Psychiatry and Psychotherapy, CCM, Mind and Brain ResearchBerlin, Germany; ^2^Independent ResearcherMaastricht, Netherlands

**Keywords:** psychiatric neurosurgery, radiosurgery, gamma knife, DBS, ablative neurosurgery, cingulotomy, capsulotomy, neuroethics

## Abstract

In the wake of deep brain stimulation (DBS) development, ablative neurosurgical procedures are seeing a comeback, although they had been discredited and nearly completely abandoned in the 1970s because of their unethical practice. Modern stereotactic ablative procedures as thermal or radiofrequency ablation, and particularly radiosurgery (e.g., Gamma Knife) are much safer than the historical procedures, so that a re-evaluation of this technique is required. The different approaches of modern psychiatric neurosurgery refer to different paradigms: microsurgical ablative procedures is based on the paradigm ‘quick fix,’ radiosurgery on the paradigm ‘minimal-invasiveness,’ and DBS on the paradigm ‘adjustability.’ From a mere medical perspective, none of the procedures is absolutely superior; rather, they have different profiles of advantages and disadvantages. Therefore, individual factors are crucial in decision-making, particularly the patients’ social situation, individual preferences, and individual attitudes. The different approaches are not only rivals, but also enriching mutually. DBS is preferable for exploring new targets, which may become candidates for ablative microsurgery or radiosurgery.

## Introduction

Since 2000, there is a renaissance of neurosurgical treatments of psychiatric disorders. Many researchers and clinicians hope that modern neurosurgical approaches will be established as treatment options for a growing number of therapy-refractory psychiatric disorders. About 90% of functional neurosurgeons feel optimistic about the future of psychiatric neurosurgery (Lipsman et al., 2011; Mendelsohn et al., 2013).

Modern psychiatric neurosurgery includes DBS and ablative neurosurgical procedures (thermal or radiofrequency ablation, and radiosurgery). DBS and thermal or radiofrequency ablation procedures require a craniotomy. Radiosurgery (Gamma Knife Radiosurgery) is performed without craniotomy, mostly as an ambulant treatment. In future, high intensity focused ultrasound might become another option. The worldwide first four patients have been treated with this technique in South Korea (Na et al., 2015).

Many authors consider DBS as the most modern and superior technology, particularly because of its adjustability and high degree of reversibility. However, in the wake of DBS development, ablative neurosurgical procedures are seeing a comeback, although they had been discredited and nearly completely abandoned in the 1970s because of their frequent serious complications and their unethical practice. Since modern stereotactic ablative procedures, particularly radiosurgery are much safer and more efficient than their historical antecessors, a re-evaluation of this technique is required.

Until now, ethical discussion about non-DBS psychiatric neurosurgery is scarce, whereas psychiatric DBS is intensively discussed ethically. This blind spot in neuroethics is astonishing for several reasons: First, the fraction of ablative procedures in psychiatric neurosurgery is big: in North America, 50% of psychiatric neurosurgeons use lesioning exclusively or combined with DBS (Lipsman et al., 2011); outside of North America even 54.9% (Mendelsohn et al., 2013). Second, two expert panels have affirmed stereotactic ablative procedures as important alternatives for appropriately selected patients (Parkinsonism: Bronstein et al., 2011; psychiatric disorders: Nuttin et al., 2014). Third, a clear superiority of any procedure in all relevant aspects cannot be established. Forth, which approach is optimal, depends significantly on patients’ individual medical and non-medical properties. Fifth, the much higher costs of DBS, particularly for long-term treatment, exclude this option for the majority of patients world-wide.

Therefore, a comprehensive ethical analysis of the pros and cons of the different approaches is necessary, based on clinical facts, not on ideological prejudices. Particularly, it is not justified to characterize modern lesioning procedures as successors of historical psychosurgery, while presenting DBS as something quite different. In fact, both psychiatric DBS and modern ablative psychiatric neurosurgery are significantly improved successors of the historical psychosurgery.

## Different Paradigms

The different approaches of modern psychiatric neurosurgery refer to different paradigms: microsurgical ablative procedures is based on the paradigm ‘quick fix,’ radiosurgery on the paradigm ‘minimal-invasiveness,’ and DBS on the paradigm ‘adjustability.’

The purpose of ablative microsurgical procedures is to disconnect limbic system circuits related to different psychiatric disorders in order to enhance brain function and reduce psychiatric symptoms (Martinez-Alvarez, 2015).

Radiosurgery is usually considered as an ablative treatment. However, recent neurophysiological, radiological, and histological studies challenge this view. Radiosurgical protocols for neurological or psychiatric disorders might have differential effects on various neuronal populations and remodel the glial environment, leading to a modulation of function while preserving basic processing. Thus, modern functional radiosurgery might be based on neuromodulatory effects (Régis, 2013).

DBS has been considered as a method to produce reversible lesions. Indeed, high-frequency DBS has a similar effect as lesions, i.e., inhibition of targets that are hyperactive in psychiatric disorders. However, its mechanism of action is unclear, and several hypotheses have been put forward to explain the blocking effect of stimulation (Lévèque, 2014). Its main advantage is that the stimulation effect can be adjusted by adapting the stimulation parameters.

## Efficacy

A direct comparison of the efficacy of the different approaches is not yet possible, particularly because of the heterogeneity of the studies, the small patient numbers, and the fact that most studies are neither placebo-controlled nor double-blind. The rapid development of the methods aggravates their comparison: In psychiatric DBS, many targets (mostly overlapping for different diagnoses) are tested with different stimulation parameters. In radiosurgery, the radiation doses used decreased significantly. Randomized controlled trials would be optimal to directly compare the efficacy of the different approaches. However, this scientific standard cannot be met for practical and ethical reasons. Nevertheless, studies that directly compare different approaches with matched patients would also provide a valid efficacy comparison. In any case, this would be much better than the current practice of publishing reviews. The problem with most reviews is that they summarize only data published in medical journals in English language. However, this practice does not represent the clinical reality but presents a distorted picture. Therefore, we expect a severe publication bias (Schläpfer and Fins, 2010), leading to a systematic over-evaluation of the benefits.

The publication bias is no minor problem in psychiatric neurosurgery, but a fundamental problem, which corrupts the evaluation of risks and benefits of the different procedures. For example, we have performed a systematic literature search on psychiatric neurosurgery for treating anorexia nervosa, which yielded only 27 cases (Müller et al., forthcoming). However, from presentations on conferences we learned that a multiple of the patients reported in journals have been treated with ablative neurosurgery. Websites of private clinics in Europe as well as in Asia offer ablative surgery for a broad spectrum of psychiatric disorders as part of clinical routine. These treatments are not part of clinical studies and usually not published. Recently, a book of Sun and De Salles (2015) has been published which presents original data from several studies with ablative neurosurgery for different psychiatric disorders which had not been published in medical journals.

That being said, we summarize available data on the efficacy of the different approaches, whereby we refer to the most recent reviews as well as to the above mentioned book of Sun and De Salles.

### Deep Brain Stimulation

For OCD, data from 25 papers comprising 109 patients and five targets (NAcc, VC/VS, ITP, nucleus subthalamicus, and internal capsule) have been published (Kohl et al., 2014). The responder rates ranged from 45.5 to 100%.

For depression, data from 22 papers comprising 188 patients and six targets (NAcc, VC/VS, SCC, lateral habenula, ITP, and slMFB) have been published (Morishita et al., 2014). The responder rates ranged from 29 to 92%. However, two multicenter, randomized, controlled, prospective studies evaluating the efficacy of VC/VS, and SCC DBS were recently discontinued because of inefficacy based on futility analyses (Morishita et al., 2014). The failure of two high quality studies in spite of the universally positive results of reported open-label trials could be attributable to the typical overestimation of efficacy associated with open label trials that arises from the failure to control for placebo, and biases due to lack of blinding and randomization (Morishita et al., 2014).

For anorexia nervosa, six papers comprising 18 patients and three targets (NAcc, subcallosal cingulum, and VC/VS) have been published (Müller et al., forthcoming). Remission (normalized body mass index) occurred in 61% of patients, and in 88.9%, psychiatric comorbidities improved, too. However, Sun et al. (2015) have recently published less favorable results: only 20% (3/15) of their patients treated with NAcc DBS showed improvements in symptoms. The other 80% underwent a second surgery (anterior capsulotomy), which improved eating behavior and psychiatric symptoms in all patients (Sun et al., 2015).

Generally, the current knowledge does not allow for identifying a superior target (Kohl et al., 2014; Morishita et al., 2014; Müller et al., forthcoming).

### Microsurgical Ablative Procedures

For treatment-refractory depression, 40–60% of patients responded to bilateral capsulotomy or cingulotomy performed with thermal coagulation or radiosurgery (Eljamel, 2015).

For OCD, response rates between 36 and 89% have been published (Martinez-Alvarez, 2015). Martinez-Alvarez (2015) reports own data of 100 OCD patients of whom 71% responded.

For anorexia nervosa, three papers with nine patients report a remission rate of 100%, with regard to both weight normalization and psychiatric comorbidities. Different targets were used (dorsomedial thalamus, anterior capsula, NAcc; Müller et al., forthcoming). Sun et al. (2015) report 150 patients treated with capsulotomy, of whom 85% experienced an improvement in symptoms.

### Radiosurgical Ablative Procedures

For OCD patients, a response rate of 70% has been reported in the literature (Martinez-Alvarez, 2015). Martinez-Alvarez (2015) reported a response rate of 100% in five own patients.

## Adverse Effects

### Deep Brain Stimulation

Following DBS, surgery-related, device-related, and stimulation-related side-effects have been reported. Serious adverse events during surgery were reported: seizures, intracerebral hemorrhages (in one case causing a temporary hemiparesis), a panic attack, and a cardiac air embolus (Kohl et al., 2014; Morishita et al., 2014; Müller et al., forthcoming). In anorexia nervosa patients, a high rate of severe complications have been reported: further weight loss, pancreatitis, hypophosphataemia, hypokalaemia, a refeeding delirium, an epileptic seizure during electrode programming, QT prolongation, and worsening of mood (Müller et al., forthcoming).

In several cases, superficial wound infections, inflammation, or allergic reactions occurred (Kohl et al., 2014). Device-related adverse effects comprised breaks in stimulating leads or extension wires requiring replacement, dysesthesia in the subclavicular region, and feelings of the leads or stimulators (Kohl et al., 2014).

Stimulation-induced adverse effects comprised mood disturbances, suicidality, anxiety, panic attacks, fatigue, and hypomania, partly induced either by a change of stimulation parameters, or by battery depletion. These effects were either adjustable by parameter adaption or device exchange (Kohl et al., 2014; Morishita et al., 2014; Müller et al., forthcoming). Some DBS patients report feelings of self-estrangement (Gilbert, 2013). A great problem is the high number of suicides and suicide attempts after DBS that have been reported in eight papers (Kohl et al., 2014; Morishita et al., 2014). Further side effects include vertigo, weight loss or gain, long-lasting fatigue, an increased headache frequency, and visual disturbance (Kohl et al., 2014).

### Microsurgical Ablative Procedures

Adverse side effects of microsurgical ablative surgery for major depression comprised epilepsy (up to 10%), incontinence, weight gain, transient confusion, transient mania, and transient incontinence. Further side effects reported by only one or two studies are personality change (7 and 10%), lethargy, hemiplegia (0.3%), and suicide (1 and 9%) (Eljamel, 2015). Following microsurgical ablative surgery for treating OCD, a similar spectrum of adverse effects has been published. Most side effects were transient, and included headaches, urinary incontinence, impaired cognitive function, and confusion. Tardive epileptic seizures occurred in 2–9% of patients (Martinez-Alvarez, 2015). In case of anorexia nervosa, the journal papers reported only transient adverse effects: bradycardia, mild disorientation, moderate somnolence, loss of concentration, apathy, emotional emptiness and mild loss of decorum, headaches, and centric fever (Müller et al., forthcoming). However, Sun et al. (2015) report intracranial hematomas in 1.9% of the patients (4/216); one patient died thereof (0.5%).

### Radiosurgical Ablative Procedures

Side-effects such as fatigue, weight gain, or apathy occurred in several patients who had received doses of more than 180 Gy. In newer studies with lower radiation doses, adverse effects did not occur (Lévèque, 2014).

## Recommendations

From a mere medical perspective, none of the procedures is absolutely superior; rather, they have different profiles of advantages and disadvantages (see **Table 1**). The main advantages of DBS are its adaptability and high degree of reversibility; of microsurgical ablative procedures the rapid onset of action; and of radiosurgery its noninvasiveness and low rate of adverse effects. Furthermore, it differs individually what counts as an advantage or disadvantage: For example, the delayed onset of action of radiosurgery makes it disadvantageous for patients who need a rapid symptom reduction. However, the gradual development of effects might be advantageous since it alleviates the psychological adjustment (Lindquist et al., 1991). This may be protective against feelings of being manipulated, self-estrangement and the burden of normality syndrome.

**Table 1:** Comparison of different approaches of modern psychiatric neurosurgery.

	DBS	Microsurgery	Radiosurgery
Paradigm	Adjustability	Quick fix	Minimal-invasiveness
Adjustability	Very high	Low (through a second intervention to produce another lesion or to enlarge the lesion)	Low (second intervention to produce another lesion) to medium (through a step-by-step approach)
Addressing different targets in a single session	No	Yes	Yes
Reversibility	High (exception: permanent adverse effects due to lesions, infections, bleeding)	No	No
Invasive craniotomy	Yes	Yes	No
Onset of action	Hours to 12 months	Days or weeks	6–12 months
Appropriateness for patients with special needs	No	Patients who would not comply with long-term follow-up	Patients - Who would not comply with long-term follow-up - With higher risks of anesthesia - With higher infection risks
Time and effort of the procedure	Single surgery; several days in hospital plus visits for adapting stimulation parameters	Single surgery; several days in hospital	Ambulatory treatment, single session
Long-term treatment	Frequent consultation of specialists required (parameter adjustment, device exchange)	Not necessary	Not necessary
Costs	Very high direct and life-long costs	Medium	Low
Mortality risk	Yes	Yes	No
Short-term risks	- Anesthesia - Infection - Hemorrhage - Hardware complications	- Anesthesia - Infection - Hemorrhage	- Development of cysts - Edemas
Long-term risks	- Infection risks (due to biofilms and regular battery exchange) - Hardware complications	No	No
Possible adverse effects	- Suicidality - Mood disturbance - Anxiety - Panic attacks - Hypomania - Weight loss or gain - Long-lasting fatigue - Increased headache frequency - Visual disturbance	- Suicidality - Headaches - Seizures - Drowsiness - Urinary incontinence Cognitive impairment - Personality change	- Transient cognitive impairment - Transient apathy - Radiation dose >180 Gy: fatigue, weight gain, or apathy
Disadvantages in daily life	Device-related problems in daily life (e.g., at airport controls)	No	No
Disadvantages for further medical treatment	- Exclusion of electroconvulsive therapy - Special MRI required	No	No
Possible problems of psychosocial adaptation	Self-estrangement, feeling of being manipulated; burden of normality syndrome	Burden of normality syndrome	Improbable

We support further research in this area generally, but think that therapeutic adventurism cannot be justified. The current research practice in psychiatric neurosurgery does not fulfill the highest ethical and scientific standards. We plead for ethical reasons for better safeguards in research and clinical practice. Since psychiatric neurosurgery has both the goal and the potential to change core features of the patients’ personalities, these interventions require a solid scientific fundament. Particularly, we recommend the following:

• Case registries should become obligatory for all clinical studies in order to avoid a publication bias and its negative consequences, namely faulty evaluations of therapies, flawed therapy recommendations, unpromising treatment attempts and unneeded clinical studies (Morishita et al., 2014). Individual treatment attempts should not be performed.
• A multi-center, randomized, controlled study should be performed that directly compares DBS, microsurgical ablative procedures and radiosurgery for different psychiatric disorders.
• Since multiple circuits seem to be involved in psychiatric disorders, targets of DBS or ablative procedures, respectively, should be selected specifically with regard to the prominent symptoms instead of using the institution-specific target for all patients.
• Since no single procedure is absolutely superior, patients should be informed comprehensively about the different treatment options and their respective benefit-risk-profiles. Individual factors have to be crucial in decision making, particularly the patients’ social situation, individual preferences, and individual attitudes (e.g., whether they could tolerate implanted devices; whether they are more afraid of the irreversibility of an ablative procedure or of the medical risks of brain surgery).

We are convinced that the different approaches are not only rivals, but also enriching mutually. DBS is preferable for exploring new targets, which may become candidates for ablative microsurgery or radiosurgery.

## Conflict of Interest Statement

The authors declare that the research was conducted in the absence of any commercial or financial relationships that could be construed as a potential conflict of interest.

## Abbreviations

ALIC: anterior limb of the internal capsule
DBS: deep brain stimulation
ITP: inferior thalamic peduncule
MRI: magnetic resonance imaging
NAcc: nucleus accumbens
OCD: obsessive-compulsive disorder
SCC: subgenual cingulate cortex
slMFB: superolateral medial forebrain bundle
VC/VS: ventral capsula/ventral striatum.
